# Supplementary material for: Modification of Regulatory T Cell Epitopes Promotes Effector T Cell Responses to Aspartyl/Asparaginyl β-Hydroxylase
Source: Int J Mol Sci. 2022 Oct 18;23(20):12444. doi: 10.3390/ijms232012444 (PMC9604227; doi:10.3390/ijms232012444)
Supplement: Supplementary file 1 [file ijms-23-12444-s001.zip › ijms-1867599-supplementary.pdf]

Supplementary Material

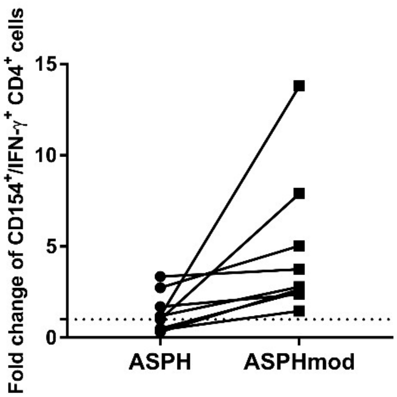

Figure S1. ASPHmod-pulsed moDCs induce an increase in CD4$^+$CD154$^+$IFN-γ^+ T cells. Relative T cell response of eight individuals to ASPH- versus ASPHmod-pulsed moDCs

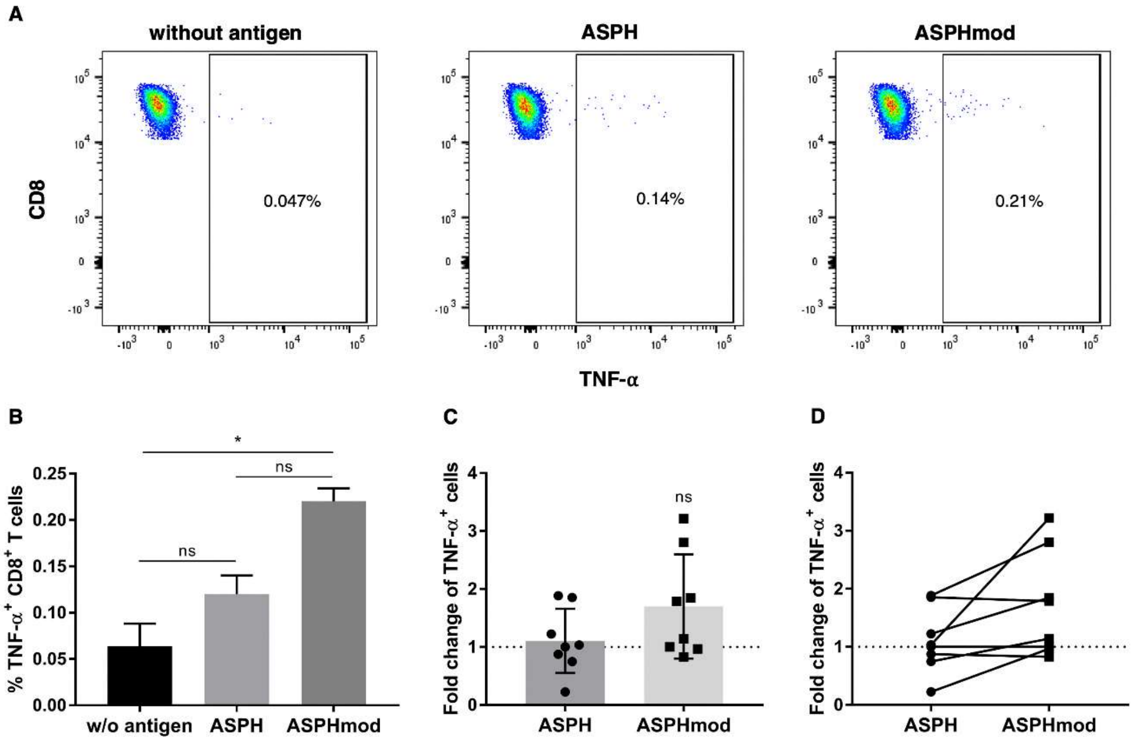

Figure S2. ASPHmod-pulsed moDCs stimulate an increased number of CD8$^+$TNF-α^+ T cells. (A) Representative flow cytometric analysis of the T cells derived from a single individual after restimulation with non-pulsed, ASPH- or ASPHmod-pulsed moDCs. (B) Data represent the means ± SD of the same individual assessed in triplicates: not significant (ns), *significantly different, $P < 0.05$ (Kruskal-Wallis test). (C) Fold change in CD8$^+$TNF-α^+ T cells relative to cells restimulated with the non-pulsed moDC control. Data

represent the means ± SD of eight individuals: not significant (ns). (D) Relative T cell responses of each individual to ASPH- versus ASPHmod-pulsed moDCs.

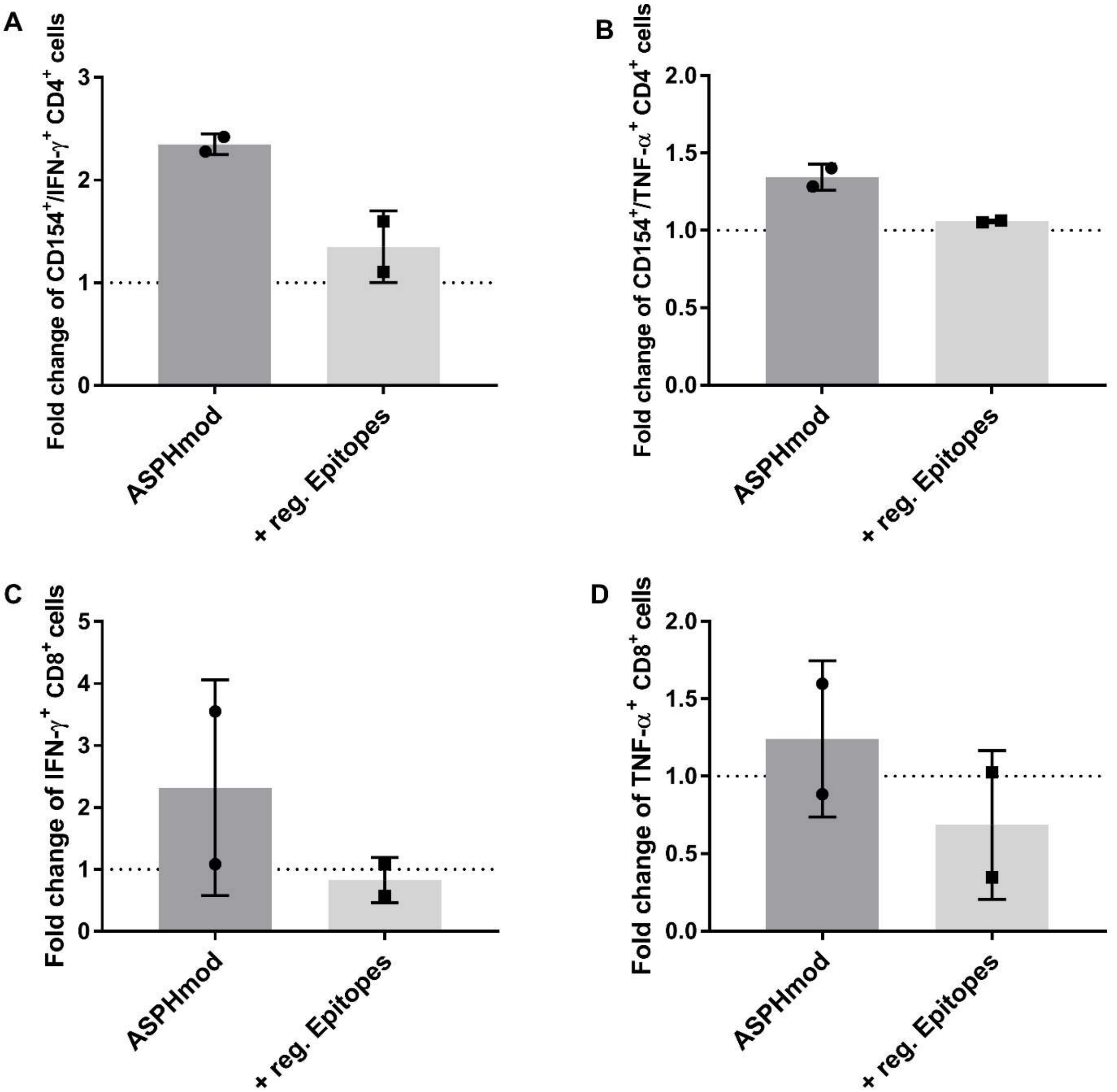

Figure S3. Adding wild type, regulatory epitopes abrogates the increased immunogenicity of ASPHmod. Fold change in (A) $CD4^+CD154^+IFN\text{-}\gamma^+$ T cells, (B) $CD4^+CD154^+TNF\text{-}\alpha^+$ T cells, (C) $CD8^+IFN\text{-}\gamma^+$ T cells and (D) $CD8^+TNF\text{-}\alpha^+$ T cells relative to cells restimulated with the non-pulsed moDC control. Data represent the means ± SD of two individuals.

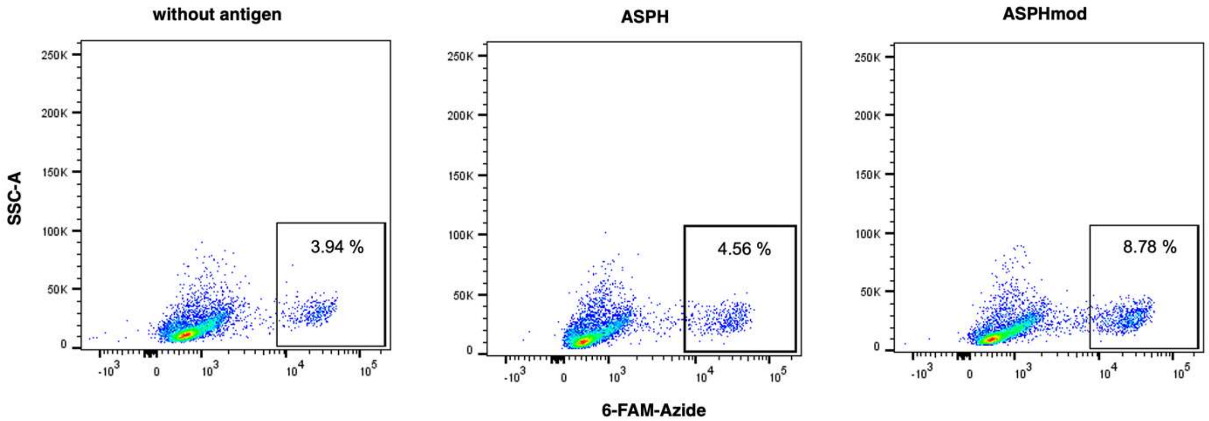

Figure S4. Analysis of T cell proliferation after coculture with non-pulsed, ASPH- and ASPHmod-pulsed moDCs. Representative flow cytometric analysis of T cell proliferation derived from a single individual.
